# Gender Differences in the Risk of HIV Infection among Persons Reporting Abstinence, Monogamy, and Multiple Sexual Partners in Northern Tanzania

**DOI:** 10.1371/journal.pone.0003075

**Published:** 2008-08-27

**Authors:** Keren Z. Landman, Jan Ostermann, John A. Crump, Anna Mgonja, Meghan K. Mayhood, Dafrosa K. Itemba, Alison C. Tribble, Evaline M. Ndosi, Helen Y. Chu, John F. Shao, John A. Bartlett, Nathan M. Thielman

**Affiliations:** 1 Duke University Medical Center, Durham, North Carolina, United States of America; 2 Duke Global Health Institute, Duke University, Durham, North Carolina, United States of America; 3 Kilimanjaro Christian Medical College, Tumaini University, Moshi, Tanzania; 4 Kilimanjaro Christian Medical Centre, Moshi, Tanzania; 5 KIWAKKUKI (Women Against AIDS in Kilimanjaro), Moshi, Tanzania; University of Cape Town, South Africa

## Abstract

**Background:**

Monogamy, together with abstinence, partner reduction, and condom use, is widely advocated as a key behavioral strategy to prevent HIV infection in sub-Saharan Africa. We examined the association between the number of sexual partners and the risk of HIV seropositivity among men and women presenting for HIV voluntary counseling and testing (VCT) in northern Tanzania.

**Methodology/ Principal Findings:**

Clients presenting for HIV VCT at a community-based AIDS service organization in Moshi, Tanzania were surveyed between November 2003 and December 2007. Data on sociodemographic characteristics, reasons for testing, sexual behaviors, and symptoms were collected. Men and women were categorized by number of lifetime sexual partners, and rates of seropositivity were reported by category. Factors associated with HIV seropositivity among monogamous males and females were identified by a multivariate logistic regression model. Of 6,549 clients, 3,607 (55%) were female, and the median age was 30 years (IQR 24–40). 939 (25%) females and 293 (10%) males (p<0.0001) were HIV seropositive. Among 1,244 (34%) monogamous females and 423 (14%) monogamous males, the risk of HIV infection was 19% and 4%, respectively (p<0.0001). The risk increased monotonically with additional partners up to 45% (p<0.001) and 15% (p<0.001) for women and men, respectively with 5 or more partners. In multivariate analysis, HIV seropositivity among monogamous women was most strongly associated with age (p<0.0001), lower education (p<0.004), and reporting a partner with other partners (p = 0.015). Only age was a significant risk factor for monogamous men (p = 0.0004).

**Interpretation:**

Among women presenting for VCT, the number of partners is strongly associated with rates of seropositivity; however, even women reporting lifetime monogamy have a high risk for HIV infection. Partner reduction should be coupled with efforts to place tools in the hands of sexually active women to reduce their risk of contracting HIV.

## Introduction

Monogamy has been increasingly advocated as one of the cornerstones of HIV prevention in sub-Saharan Africa. As part of the “ABC” approach—urging abstinence, being monogamous, and using condoms—this strategy has been associated with reduced transmission of HIV among sexually active people both in epidemiological modeling studies [1] and in other research in developing countries [2], [3].

Differences between dynamics of female-to-male and male-to-female HIV transmission [4] and between trends in sexual behavior among men and women in the developing world [5], [6] suggest that sexual behavior may differentially impact transmission risk among men and women. In one Zimbabwean cohort, for instance, the risk of HIV rose with increasing number of sexual partners among women, but not among men [6].

We examined the association between the number of lifetime sexual partners and the risk of HIV seropositivity among men and women presenting for HIV voluntary counseling and testing (VCT) in northern Tanzania and further identified risk factors for seropositivity among monogamous women and men in this cohort.

## Methods

### Study population and Data Collection

Study subjects were recruited at a free-standing VCT center operated by Kikundi cha Wanawake Kilimanjaro Kupambana na UKIMWI (KIWAKKUKI; Women Against AIDS in Kilimanjaro), a women-led HIV/AIDS advocacy, education, and home care organization based in Moshi, Tanzania that provides services for men and women. Free VCT services are offered to anyone requesting HIV testing at this free-standing walk-in facility, located one block from a busy regional bus station; the majority of clients seen are self-referred. With the exception of a 3 week national VCT campaign period in October 2007, all clients age 18 or older were approached consecutively for study participation and refusal rates were < 5%. At the time of data collection, KIWAKKUKI's VCT center saw an average of 13 clients each weekday [7], [8].

After obtaining informed consent, clients presenting for VCT between November 2003 and December 2007 were interviewed by trained, Tanzanian Kiswahili-speaking counselors using a standardized questionnaire developed for this project. The questionnaire gathered data on sociodemographic characteristics, reasons for testing, and sexual behavior, and included the questions, “How many sexual partners have you ever had in your lifetime?” and “Do you have a sexual partner who has other partners?” In addition, trained VCT counselors asked if clients presented for testing because of illness and if they had symptoms commonly associated with HIV infection in this population (recent fever, cough, hemoptysis, night sweats, diarrhea, genital ulcers/discharge, rash, or weight loss) [8]. Client response data were recorded on paper questionnaires by the counselors.

### Statistical analyses

Data from unique records of first-time clients aged 18 and over were entered into a Microsoft Access (Redmond, Washington, USA) sing either EpiInfo 3.3 (CDC, Atlanta, GA, USA) or Teleform 9.0 (Cardiff, Visa, CA) and analyzed using STATA 10 (StataCorp, College Station, TX, USA). Factors associated with HIV seropositivity were identified by multivariate logistic regression models.

Men and women were categorized by the number of lifetime sexual partners reported, and rates of HIV seropositivity were calculated for categories of subjects reporting 0, 1, 2, 3 or 4, and 5 or more partners. Subjects with 3 or 4 reported lifetime partners, and those with 5 or more partners, respectively, were grouped due to lower numbers in these categories. These categories are identical to those used in a similar analysis by other investigators [6]. We compared trends in HIV seropositivity by number of sexual partners, and across various demographic and behavioral characteristics using Chi square and nonparametric trend tests [9].

To derive an estimate of the annualized risk of HIV infection, we divided the number of positive tests by the total exposure time. A person's exposure time was defined as the time between the age at sexual debut and the age at the test date, accounting for estimates that the HIV epidemic in Tanzania began in 1983 [10]. To assess the degree to which demographic correlates and having a partner with other partners (partner concurrency) affect the risk of seropositivity, we estimated multivariate logistic regression models separately for each gender, and separately for all respondents and for monogamous respondents only. In sensitivity analysis, we conducted stratified analyses for specific population sub-groups. To assess whether selection bias could explain gender differences in seroprevalence, we examined trends in a group comprised of only asymptomatic subjects.

### Research ethics

The protocol for this study was approved by the KCMC Research Ethics Committee, the Tanzania National Institutes for Medical Research National Research Ethics Coordinating Committee, and an Institutional Review Board of Duke University Medical Center. All study participants provided written informed consent prior to data collection.

## Results

Of 6,549 clients with at least one lifetime partner, 3,607 (55%) were female. The mean (median; range) age of males was 33 (29; IQR 24–40) years and that of females was 32 (30; IQR 24–40) years (*p* = 0.8384). Nine hundred and thirty-nine (25%) females were HIV seropositive, whereas 293 (10%) males were seropositive (p<0.0001). Four hundred and twenty-three (14%) males and 1,244 (34%) females reported having had only one lifetime sexual partner (*p*<0.0001). Women having more than one lifetime sexual partner reported fewer total partners (median 3, IQR 2–4) than similar men did (median 4, IQR 3–7, p<0.0001). After excluding clients missing information on sexual debut or on covariates, 6,104 respondents were included in the multivariate analyses.

**Figure 1:**
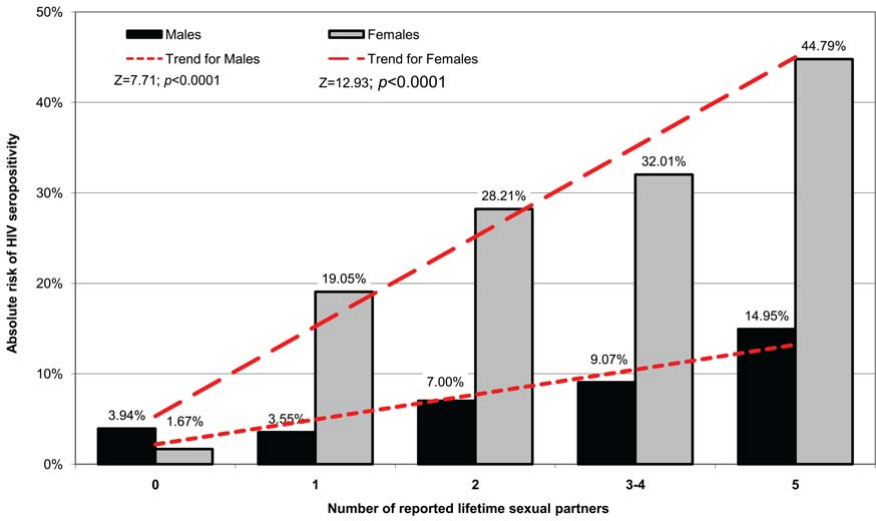
Absolute risk of HIV seropositivity among women and men presenting for VCT, by number of lifetime sexual partners in Moshi, Tanzania, 2003–2007, N = 6,531.

As expected, rates of HIV were lowest in abstainers and highest among persons reporting 5 or more sexual partners. The absolute risk of HIV seropositivity increased monotonically with the number of lifetime sexual partners in both men and women. Among respondents with at least one sexual partner, the absolute risk (Figure 1) for HIV seropositivity was higher for women than for men; the risk for women relative to men within each subgroup ranged from 3.0 to 5.4. A trend test of increasing rates of seropositivity with increasing numbers of partners was significant for both men (Z = 7.71, p<0.0001) and women (Z = 12.93, p<0.0001, Figure 1).

**Figure 2:**
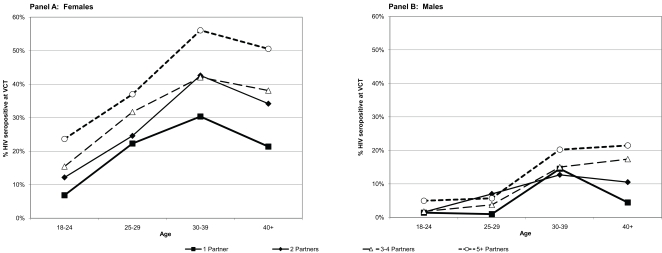
HIV seropositivity among women and men presenting for VCT, by number of lifetime partners and age of tester in Moshi, Tanzania, 2003–2007. Among women, subjects between 30 and 39 years old had the highest risk of seropositivity in each category of lifetime sexual partners (Figure 2, Panel A), while subjects 40 years or older had the greatest rise in risk of seropositivity with increasing numbers of sexual partners. The associations were similar but not as strong among men (Figure 2, Panel B). Non-parametric trend tests of associations between the number of partners and seropositivity were significant among men in the youngest and two oldest age groups only (p = 0.016; p = 0.035; and p = 0.001, respectively) and among women showed significant effects in all age groups (p<0.0001 to p = 0.006).

Among women, subjects between 30 and 39 years old had the highest risk of seropositivity in each category of lifetime sexual partners (Figure 2), while subjects 40 years or older had the greatest rise in risk of seropositivity with increasing numbers of sexual partners. The associations were similar but not as strong among men (data not shown): non-parametric trend tests of associations between the number of partners and seropositivity were significant among men in the youngest and two oldest age groups only (p = 0.016; p = 0.035; and p = 0.001, respectively) and among women showed significant effects in all age groups (p<0.0001 to p = 0.006). Accounting for the beginning of the HIV epidemic in Tanzania in 1983, and the time between subjects' sexual debut and their age at testing (i.e., the exposure time), the annualized risk per 100 person years for monogamous males was 0.51 (95% confidence interval (CI), 0.30–0.86) versus 1.67 (95% CI, 1.47–1.90) for monogamous females, and the risk increased within each category with increasing numbers of sexual partners (Table 1).

**Table 1:** Annualized rates of HIV infection among male and female VCT clients in Moshi, Tanzania, 2003–2007.

Number of Partners	Males				Females			
	Person-years at risk	Number HIV-infected	Rate*	95% Conf. Limits	Person-years at risk	Number HIV-infected	Rate*	95% Conf. Limits
1	2,754	14	0.51	0.30–0.86	14,091	236	1.67	1.47–1.90
2	5,093	36	0.71	0.51–0.98	12,127	276	2.28	2.02–2.56
3–4	9,055	71	0.78	0.62–0.99	10,587	256	2.42	2.14–2.73
5 or more	15,798	159	1.01	0.86–1.18	4,834	145	3.00	2.55–3.53
Total	32,700	280	0.86	0.96–1.18	41,639	913	2.19	2.05–2.34

* Rates of HIV infection per 100 person years at risk

In multivariate analysis, a greater number of sexual partners was associated with seropositivity in women, and to a somewhat lesser extent in men (Table 2). Age was strongly predictive of seropositivity. Females ages 30–39 and males ages 30 and above were most likely to test seropositive. Being widowed and having weekly household expenditures below 7000 Tanzania shillings (TSH; approximately 5.80 United States dollars) were associated with seropositivity in both men and women, while post-secondary education was protective. Having a partner who had other sexual partners or not knowing whether the partner had other partners was associated with seropositivity among monogamous females (odds ratio 1.44, 95% CI, 1.07–1.97) but was not associated with seropositivity in men.

**Table 2:** Correlates of HIV infection by gender among 6,104 clients presenting for VCT in Moshi, Tanzania, 2003–2007.

	Percent	Females	Males	Monogamous	Monogamous
				Females	Males
1 Partner	26.9	*ref.*	*ref.*		
2 Partners	24.5	1.77*** [1.43;2.19]	1.56 [0.83;2.95]		
3–4 Partners	25.9	2.20*** [1.77;2.75]	1.89* [1.05;3.41]		
5+ Partners	22.7	3.44*** [2.60;4.55]	2.75*** [1.56;4.83]		
Age 18–24	28.7	*ref.*	*ref.*	*ref.*	*ref.*
Age 25–29	20.0	2.51*** [1.90;3.31]	1.78 [0.98;3.22]	3.93*** [2.31;6.70]	0.72 [0.07;7.01]
Age 30–39	27.3	3.96*** [3.07;5.10]	6.63*** [4.00;11.00]	5.87*** [3.64;9.47]	15.63 [3.73;65.51]
Age 40+	24.0	2.36*** [1.80;3.10]	5.67*** [3.36;9.58]	3.31*** [2.02;5.42]	4.85 [0.69;34.03]
Widowed	10.1	2.75*** [2.22;3.41]	2.96*** [1.85;4.73]		
Post-secondary education	18.6	0.62** [0.47;0.82]	0.54** [0.37;0.78]	0.45** [0.26;0.77]	1.82 [0.49;6.74]
Town	46.8	0.94 [0.79;1.12]	0.80 [0.61;1.05]	0.90 [0.65;1.25]	1.54 [0.50;4.78]
Household expenditures: < TSH 7,000 / week	35.6	1.27** [1.07;1.51]	1.33* [1.01;1.77]	1.52** [1.13;2.06]	1.50 [0.48;4.62]
Partner with other partners - Yes or don't know	44.1	1.16 [0.98;1.37]	1.15 [0.88;1.50]	1.44* [1.07;1.94]	1.01 [0.33;3.05]
(N)		(3334)	(2770)	(1227)	(415)

Odds ratios and [95% confidence intervals] from logistic regression models predicting seropositivity. ^*^, ^**^, and ^***^ denote statistical significance at the 0.05, 0.01, and 0.001 levels, respectively. Ref. denotes reference category. Observations with missing covariates were dropped from the analysis. TSH, Tanzania shilling.

When assessing HIV seroprevalence in a monogamous population, the partner's risk behaviors are of particular interest. We used parameter estimates from the logistic regression model to calculate the marginal effect of monogamous women's partners having other partners on rates of seropositivity. A monogamous female with a partner who has other partners or who does not know if the partner has other partners is 36% more likely (CI, 7% to 65%, p = 0.016) to be HIV-infected than an otherwise identical female who reports no partners with other partners.

### Sensitivity Analysis

To examine if the gender difference in seropositivity could be explained by high-risk women being more likely to present for testing, we assessed how the HIV risk ratio between women and men varied in more homogenous sub-populations (e.g., age 20–30; not widowed; or no partners with other partners). Despite significant differences in the gender distribution of these sociodemographic risk characteristics, gender differences in rates of seropositivity remained large and statistically significant in all comparisons (p = 0.01 to p<0.001, not shown).

A higher proportion of monogamous women (30%) than men (15%) presented with symptoms or because of illness (p<0.001). However, this difference was not significant among HIV-seropositive clients. Additionally, with restriction of the sample to asymptomatic clients, the risk of HIV infection among monogamous women remained more than 3 times as high as that of men (p<0.001; not shown). In this group, the rate of increase in the risk of HIV infection as numbers of partners increased was similar between men and women (p = 0.9567).

## Discussion

In a large cohort of VCT clients in Moshi, Tanzania, the risk for HIV infection increased monotonically with increasing numbers of clients' sexual partners. The rate of increase was higher among women than among men, and women reporting lifetime monogamy had a significantly higher risk for HIV infection than monogamous men. These findings demonstrate limited protection of monogamy among women and highlight the risk of partner concurrency.

Epidemiological models have suggested that having concurrent sexual partnerships increases the risk for transmission of viral sexually transmitted infections (STI's), including HIV [1], [11]. Supporting these models, several studies in the United States have demonstrated concurrency and unawareness of partners' concurrency to be risk factors for transmission of syphilis and viral STI's [12], [13]. While several reports from sub-Saharan Africa have been unable to demonstrate a link between concurrency and the spread of HIV [14], [15], we found significantly higher rates of HIV infection among women reporting certain or possibly polygynous partners. Such women were 36% more likely to be HIV-infected than otherwise identical women reporting that their partner had no other partners.

Our study had two primary limitations. First, the study population was comprised entirely of VCT service users, potentially limiting the generalizability of the findings to the general population. To explore the validity of the observed gender differentials in HIV seroprevalence we considered two alternative explanations. One possibility was a differential distribution of demographic correlates of HIV infection by gender, the other a possible selection bias resulting from gender differences in the probability of presenting for VCT only after becoming symptomatic. Results from multivariate analyses suggest that differences in demographic or selected risk characteristics are not likely to be the primary explanations for the gender differences in rates of seropositivity. The differential risk of HIV seropositivity in women relative to men was consistent across demographic subgroups, and persisted regardless of the presence of risk factors. Similar trends were observed in all subgroups, including only asymptomatic clients, suggesting that self-selection for testing was not likely a primary explanation for the higher burden of HIV seropositivity among the women.

The second limitation is the study's reliance on self-reporting, which may have resulted in inaccurate measurement of risk factors. Specifically, one potential explanation for higher HIV seroprevalence among female subjects was that female subjects underreported their sexual behaviors. This possibility is not easy to rule out. In many parts of sub-Saharan Africa, the belief that women's sexual needs are not as strong as men's and the widespread, socially acceptable practice of polygyny have been associated with underreporting of extramarital sexual activity by women [16]–[18]. One study of rural Tanzanians estimated that single women underreport their numbers of sexual partners by 46% [18]. In men, the evidence is mixed, with studies suggesting underreporting of early sexual debut among young Zambian men [5] and over reporting of extramarital sexual activity in Tanzania [18]. The magnitude of the difference in HIV seroprevalence between men and women in Figure 1 (men with 5 or more partners have a lower rate of HIV infection than monogamous women) suggests that differential underreporting of sexual exposures by women is not likely a primary explanation. If women consistently misrepresented sexual exposures, the association between exposure and seroprevalence would likely resemble a flatter line or a concave curve; the highly significant and nearly linear trend in Figure 1 for females (p<0.0001) argues for the validity of reported differentials in women's sexual exposures.

The observed high rates of HIV infection in monogamous women, and the strikingly large effect of monogamous women's partners having other partners on infection rate suggest that efforts to promote abstinence, to reduce the number of sexual partners, and to promote mutual monogamy should be coupled with methods which empower women to better control their exposure risk. We recommend increased efforts to educate and empower women with respect to condom use [19], while continuing other efforts to reduce HIV transmission, including the promotion of male circumcision [20]–[22] (and further evaluation of its impact on HIV transmission risk to females) and development of vaginal microbicides [23], [24]. In a climate where many women are placed at greater risk because of partners' concurrent relationships, an overly simple formulation of the ABC prevention strategy, “if not A, then B, and if not B, then C,” is misguided. Greater means to control the risk for HIV infection should be given to all women, including those who are monogamous.
